# Genetic parameters and potential of reducing tail and ear damage in pigs through breeding

**DOI:** 10.1186/s12711-025-00976-0

**Published:** 2025-07-14

**Authors:** Bernadett Hegedűs, Natália Galoro Leite, J. Elizabeth Bolhuis, Piter Bijma

**Affiliations:** 1 Animal Breeding and Genomics, Wageningen University & Research, Droevendaalsesteeg 1, 6700 AH Wageningen, The Netherlands; 2 Adaptation Physiology, Wageningen University & Research, De Elst 1, 6708 WD Wageningen, The Netherlands; 3 Topigs Norsvin Research Centre, Meerendonkweg 25, 5216 TZ ‘s-Hertogenbosch, The Netherlands

## Abstract

**Background:**

Ear and tail biting are behaviours in pigs that cause both welfare problems and financial losses. Data collection of behaviour is difficult at the large scale needed for breeding. The damage inflicted on victims can, however, serve as a proxy for animal breeding. Here, we analysed tail and ear damage scores on their original scale, binary scale, and summed versions of these damage traits to investigate which trait definition is best for genetic selection. Using data from six purebred lines (33,329 animals in total) we aimed to (1) estimate genetic parameters for ear and tail damage using direct genetic models, (2) estimate the genetic correlation between tail and ear damage, (3) compare different trait definitions and their impact on accuracy, dispersion, and bias of estimated breeding values (EBV), and (4) compare expected responses to selection for each trait definition.

**Results:**

The heritability of the damage traits ranged from 0.04 to 0.06. Ear and tail damage were moderately correlated (0.41–0.45), meaning that the genetic propensity of being a victim is a different trait for tail versus ear biting. Estimates of the accuracy of the EBV for the traits with a five-fold cross-validation and the linear regression method based on pedigree relationships ranged from 0.27 to 0.57, the dispersion from 0.91 to 1.18, and the bias was negligible. With a selected proportion of 5%, genetic progress of ~ 0.20–0.78 genetic standard deviations per generation can be reached, depending on the trait. It was trait dependent whether direct or indirect selection yielded the most response.

**Conclusions:**

Ear and tail damage are heritable traits and are moderately positively correlated. The EBV for the evaluated traits related to ear and tail damage showed moderate accuracies, minor dispersion, and no bias. We hypothesize that from a welfare perspective, ear and tail damage on the original scale are the relevant breeding goal traits. For ear damage on the original scale, the highest response to selection can be expected when selecting on the trait itself, whereas for tail damage on the original scale, selection on summed damage showed the highest gain. Results from this study show that genetic improvement of the direct genetic effect of ear and tail damage is possible.

**Supplementary Information:**

The online version contains supplementary material available at 10.1186/s12711-025-00976-0.

## Background

Pigs rank as the second largest livestock population in the EU, entailing 141 million animals in 2021 [1], resulting in an annual production of around 23 million tonnes of meat in 2021 [2]. As most pigs are housed in groups [3], their productivity and welfare depend on social interactions between pen mates. Thus, positive and harmful interactions among pigs are an important aspect to consider, both in animal husbandry and breeding. Examples of harmful interactions are tail and ear biting, which are frequent welfare problems in group-housed pigs [4, 5].

Tail biting is the dental manipulation of another pig’s tail, which can start off gently but can later result in more severe biting. As tail biting can cause stress and wounds, it has a negative effect on the welfare of the receiver. Moreover, tail biting may also reflect an underlying welfare problem in the biter [5]. Tail biting, however, not only affects the welfare of the animals, but also results in economic losses due to reduced body weight gain, carcass condemnation, treatment cost, and even death of the victims [6–9]. Tail biting has also been associated with disease transmission and poor health [5, 10]. Many risk factors for tail biting have been identified, such as diet, stocking density, and lack of enrichment, and other factors may also play a role, such as sex and health [11, 12]. Routine tail docking as a preventive measure against tail biting is banned in the EU [3], however, it is still widely used in most EU countries [13]. This illustrates the urgent need for other preventive measures, such as deacreasing tail damage prevalence by means of genetic selection.

Data on the prevalence of tail biting behaviour on a large number of animals is difficult to obtain because recording tail biting currently requires laborious human observation. The presence of tail damage is easier to records and its reported prevalence varies largely between studies, ranging from 0.26 to 59.1% [8, 9, 14–17]. This large variation could be due to differences in the exact definition and scoring of tail damage between studies, as well as the tail length (docked vs. non-docked tails) of the pigs under study [4, 11].

Ear biting is a behaviour similar to tail biting and involves manipulation of another individual’s ear, including chewing, pulling, or quickly biting [18]. Ear biting has been associated with ear necrosis [19], which involves lesions on the pinna and may also involve infections [20]. Compared to tail biting, less information is available on ear biting behaviour and the prevalence of ear damage. Nonetheless, some studies show the importance of ear damage. In Ireland, van Staaveren et al. [21] found a median prevalence of ear lesions of 9% for the 8 to 13 week age group, and 3% for the 13 to 23 week age group. Smulders et al. [16] reported that 14.9% of the pens had at least one animal with ear damage in a sample of 3590 pens from 59 farms in Belgium.

There are only a few studies on the genetic background of tail biting behaviour [12, 22]. Breed differences were found in some studies, but not in others [12]. Breuer et al. [22] estimated the heritability to be 0.05 in Landrace pigs but found no significant heritability in Large White pigs. As tail biting and ear biting behaviours are difficult to measure on a large scale, tail and ear damage recorded on the victims are often used as proxies to study these traits. It should be noted that a classical genetic analysis of tail or ear damage only provides information on the receiver, leaving the genetic effects of the biter unidentified. Recent studies estimated the heritability of the receiver component of tail biting by analysing tail damage data using different trait definitions [23–25]. The estimated heritabilities based on linear mixed models ranged from 0.06 to 0.08 for binary tail damage in [23], was 0.09 based on tail damage medication records in [24], and was slightly higher (0.16) for tail damage on a three-point scale in [25]. For ear damage, the one available estimate of heritability is 0.46 based on a three-point scale [25]. Gorssen et al. [25] further estimated the genetic correlation between ear and tail damage to be 0.32. To our knowledge, heritability estimates of ear biting behaviour have not been reported, although between breed differences have been published [26]; observations of 100 pigs per breed showed that Landrace pigs performed less ear biting compared to Large White and Duroc pigs [26]. In addition, higher estimated breeding values for litter birth weight and test daily gain were associated with increased ear biting behaviour, suggesting genetic correlations with ear biting behaviour [27].

Knowledge of the genetic parameters of tail and ear damage and of the genetic correlation between these two traits is limited [23–25], and the two traits have been analysed using different trait definitions. However, the impact of the trait definition on genetic parameter estimates, on accuracies of estimated breeding values, and on expected responses to selection have not been reported. Therefore, the aim of this study was to (1) estimate genetic parameters for ear and tail damage using direct genetic models, resulting in genetic estimates for the recipient component of ear and tail biting, (2) estimate the direct genetic correlation between tail and ear damage, (3) compare different trait definitions and their impact on accuracy, dispersion and bias of estimated breeding values, and (4) compare expected responses to selection for each trait definition.

## Methods

## Dataset

The dataset used for this study was recorded and provided by Topigs Norsvin. The population consisted of 33,329 animals descending from 522 sires and 6656 dams. All animals were born and raised in 13 nucleus farms located in Norway, Germany, and Canada. Most farms only housed one line but three farms housed two lines. The data included three sire lines and three dam lines of Yorkshire, Large White, Landrace, Pietrain, or Duroc origin (Table 1). All pigs were fed ad libitum. Pens contained animals of a single line only. The dataset contained information on sex (intact boars vs. gilts; no barrows were included), litter (8673 levels), heard-year-season of birth (508 levels), date of birth (2021 May–2022 October), line (6 levels), litter size (min = 1, mean = 12.5, max = 30), number of pen mates at the start of the testing period (min = 6, max = 16, mean = 12.1), date when damage was scored (2021 November–2023 February), the technician who scored the damage (23 levels), age at scoring (min = 119 days, mean = 153.5 days, max = 221 days), and heard-year-season of scoring (64 levels). Most animals (32,490) were housed in groups of pigs originating from more than one litter. A total of 546 animals were kept in mixed sex groups, while the remaining pigs were in single sex groups. Mixed sex groups were not excluded from the dataset. The pedigree file used to calculate the relationship matrix (**A**) included 50,644 relationship records dating back three generations from phenotyped animals.

**Table 1:** Number of phenotyped animals by line

Line	Breed of origin	Number of phenotypes
Dam line 1	Yorkshire and Large White	5920
Dam line 2	Landrace	1391
Dam line 3	Yorkshire and Large White	11,689
Sire line 1	Duroc	1435
Sire line 2	Yorkshire and Piétrain	11,231
Sire line 3	Piétrain	1663

**Fig. 1:**
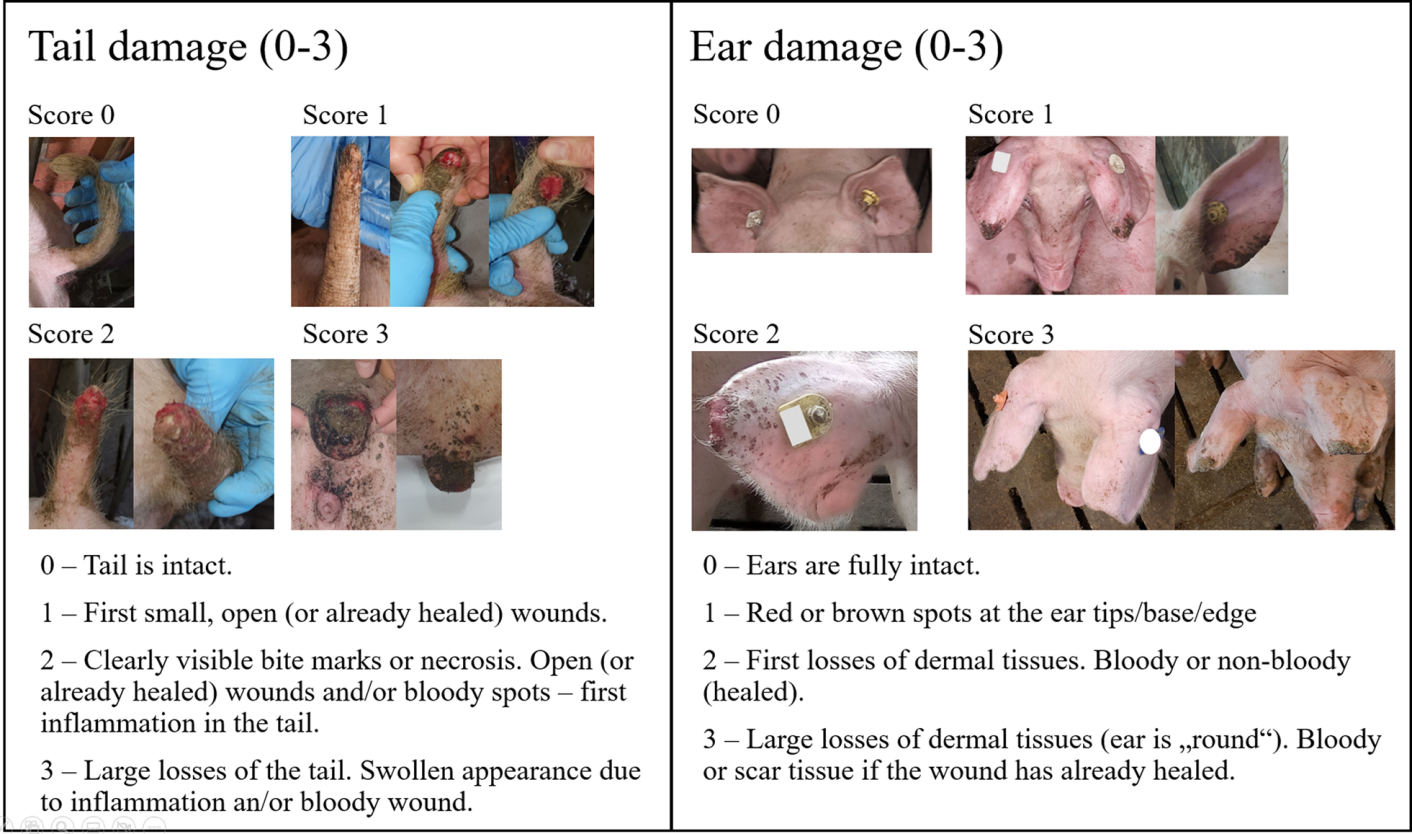
Scoring for tail and ear damage. Description of scoring of tail and ear damage. The damage score for the ears corresponds to the score of the worst ear. Note that damage resulting from biting and necrosis are not distinguished in the scoring. Pictures by Stefanie Nuphaus

**Fig. 2:**
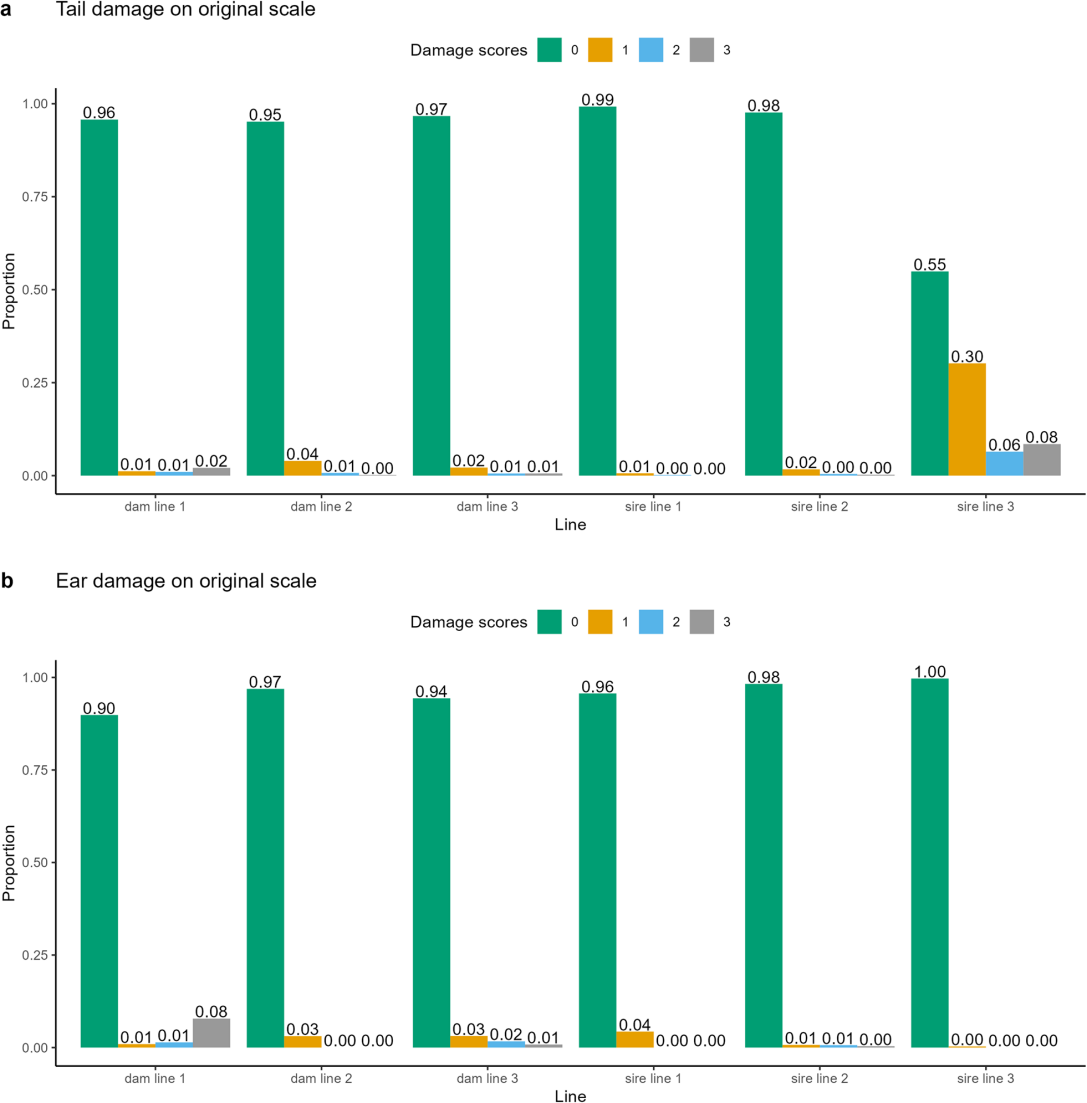
Distributions of phenotypic values. Distribution of tail (**a**) and ear damage (**b**) on the original 0 to 3 scale

The pigs were phenotyped for tail and ear damage at the end of the testing period. This was a convenient time to phenotype the damage scores as the animals were also individually weighed at this time. The range of the variable age at scoring was the result of between animal differences in reaching the goal end weight for the testing period. The animals were scored for both tail and ear damage on a scale of 0 to 3, where 0 means no damage and 3 is severe damage. There was no differentiation made between damage due to biting or due to necrosis. Figure 1 shows examples of each score. Figure 2 shows the distribution of the scores on the original scale. Compared to the other lines, sire line 3 had the highest incidence of tail damage, while dam line 1 had the highest incidence of ear damage.

## Trait definitions

The original scale of the damage traits was used to create five additional traits (Table 2). Both for ear and tail damage, a binary alternative was created (ED01 and TD01), where 0 meant no damage and damage scores of 1 to 3 were encoded as 1. A binary trait was also created for both traits together (any damage, AD01), here 0 means neither ear nor tail damage and 1 means that at least one of these body parts had damage. The trait number of body parts affected (NBP) describes no damage (0), damage on only tail or ears (1), or damage on both tail and ears (2). Finally, SD06 is the sum of the original damage scores over both traits.

**Table 2:** Trait definitions with their abbreviations

ED03	Ear damage on original scale (scale: 0–3)
TD03	Tail damage on original scale (scale: 0–3)
ED01	Binary ear damage (scale: 0–1)
TD01	Binary tail damage (scale: 0–1)
AD01	Any damage binary (scale: 0–1)
NBP	Number of body parts affected (scale: 0–2)
SD06	Sum of damage on the original scale (scale: 0–6)

## Animal model for variance component estimation

We used an animal model to estimate the genetic parameters for the different traits. We used linear models instead of threshold models as they are easier to implement in practice. We fitted all models both on the whole dataset and for each line separately, to determine whether genetic parameters differ between lines. First, the following univariate model was fitted.

1 $${\mathbf{y}} = {\mathbf{Xb}} + {\mathbf{Z}}_{{\mathbf{a}}} {\mathbf{a}} + {\mathbf{Z}}_{{\mathbf{l}}} {\mathbf{l}} + {\mathbf{Z}}_{{\mathbf{g}}} {\mathbf{g}} + {\mathbf{Z}}_{{{\mathbf{hys}}}} {\mathbf{hys}} + {\mathbf{e}}$$

where** y** is a vector of phenotypic observations for the trait, $\mathbf{b}$ is a vector of fixed effects, with incidence matrix $\mathbf{X}$, $\mathbf{a}$ is a vector of random animal effects, with incidence matrix ${\mathbf{Z}}_{\mathbf{a}}$, $\mathbf{l}$ is a vector of random litter effects, with incidence matrix **Z**_**l**_**,** $\mathbf{g}$ is a vector of random pen-group effects, with incidence matrix** Z**_**g**_**,** and **hys** is a vector of random herd-year-season effects of the time of scoring, with incidence matrix **Z**_**hys**_. The fixed effects included sex, line, the interaction of sex and line, scorer, and the age at scoring in days. Age at scoring was fit as a covariate; the other fixed effects were fit as factors. Group composition (mixed-sex vs. single-sex) was included as a fixed effect in a preliminary model but was not significant and therefore removed.

The covariance structure of the random effects in the model was assumed to be as follows:

$${\mathbf{var}} \left[ {\begin{array}{*{20}c} {\mathbf{a}} \\ {\mathbf{l}} \\ {\mathbf{g}} \\ {{\mathbf{hys}}} \\ {\mathbf{e}} \\ \end{array} } \right] = \left[ {\begin{array}{*{20}c} {{\mathbf{A}}{\upsigma }_{{\text{a}}}^{2} } & {\mathbf{0}} & {\mathbf{0}} & {\mathbf{0}} & {\mathbf{0}} \\ {\mathbf{0}} & {{\mathbf{I}}{\upsigma }_{{\text{l}}}^{2} } & {\mathbf{0}} & {\mathbf{0}} & {\mathbf{0}} \\ {\mathbf{0}} & {\mathbf{0}} & {{\mathbf{I}}{\upsigma }_{{\text{g}}}^{2} } & {\mathbf{0}} & {\mathbf{0}} \\ {\mathbf{0}} & {\mathbf{0}} & {\mathbf{0}} & {{\mathbf{I}}{\upsigma }_{{{\text{hys}}}}^{2} } & {\mathbf{0}} \\ {\mathbf{0}} & {\mathbf{0}} & {\mathbf{0}} & {\mathbf{0}} & {{\mathbf{I}}{\upsigma }_{{\text{e}}}^{2} } \\ \end{array} } \right],$$

where $\mathbf{A}$ is the relationship matrix based on pedigree*,* ${\upsigma }_{\text{a}}^{2}$ is the additive genetic variance, $\mathbf{I}$ is an identity matrix, ${\upsigma }_{\text{l}}^{2}$ is the variance of the litter effects, ${\upsigma }_{\text{g}}^{2}$ is the variance of the group effects, ${\upsigma }_{\text{hys}}^{2}$ is the variance of the heard-year-season effects of scoring, and ${\upsigma }_{\text{e}}^{2}$ is the residual variance. The pedigree did not contain across-line relationships.

Next, bivariate models were used for all pairwise trait combinations to estimate genetic correlations. The initial values for the variance components for these models were the results from the univariate models. The fixed and the random effects were the same as in the univariate models.

We fitted the models in ASReml-R version 4.1 [28]. The analysis did not converge and the correlation between the litter effects was close to one for two bivariate models (TD01-TD03 and NBP-AD01) when using the whole dataset. For these analyses, we used ASReml-SA 4.2 [29] and fixed the litter correlation at 0.99, which solved the convergence issue. This was necessary as the two traits were too similar and the genetic correlation was not estimable without fixing the litter correlation.

## Comparison of trait definitions

### Validation—accuracy, dispersion, and bias

We estimated different validation metrics to compare the trait definitions. We estimated the accuracies of EBV based on two types of cross-validations and on model derived standard errors. The last method was added to see whether the accuracies of individual animals, i.e. model derived accuracies, give similar results to cross-validation methods.

#### Model derived accuracy

The accuracy of the estimated breeding values (EBV) was calculated based on the standard error derived for the BLUP by ASReml-R Eq. (2):

2 $$r_{i} = \sqrt {1 - \frac{{{\text{PEV}}\left( {{\hat{\text{a}}}_{i} } \right)}}{{\widehat{{{\upsigma }_{{\text{a}}}^{2} }}}}}$$

where $\text{PEV}({\widehat{\text{a}}}_{i})$ is the prediction error variance of an individual pig’s EBV (i.e., the square of the standard error reported by ASReml-R [28]) and $\widehat{{\upsigma }_{\text{a}}^{2}}$ is the estimated additive genetic variance. The model derived accuracy was calculated for each phenotyped animal and each trait. In the results section, we will report averages over animals and the distribution of accuracies for each trait definition.

#### Five-fold cross-validation

Accuracy and dispersion of the EBVs were estimated based on a five-fold cross-validation using univariate models. The dataset of the phenotypes of all lines was divided into five sets by randomly allocating all records to one of five groups. Two additional masking strategies were also used: one where full-sibs were always in the same group (FS scenario) and one where half-sibs were always in the same group (HS scenario). The data from four groups (prediction set) were used to predict the breeding values of the remaining group (validation set), and this was replicated five times, using each group once as the validation set. Allocation of individuals to groups was replicated 100 times, so all together we had 100 replicates of five-fold cross-validation for each scenario. For each scenario, the mean accuracy and dispersion were saved for each of the 500 cross-validations.

Accuracy of EBVs in each validation data set was estimated as

3 $${\uprho }_{{{\hat{\text{a}}},{\text{a}}}} = \frac{{{\uprho }_{{{\text{y}}_{{{\text{v}},{ }}} {\hat{\text{a}}}_{{\text{v}}} }} }}{{\text{h}}}$$

where $\widehat{\text{a}}$ is the EBV, $\text{a}$ is the true breeding value, ${\uprho }_{{\text{y}}_{\text{v}, }{\widehat{\text{a}}}_{\text{v}}}$ is the correlation between adjusted phenotypes and EBV of the validation individuals, and h is the square root of the heritability estimated from the univariate model. For this purpose, the phenotypes of the validation set were adjusted for all fixed and random effects, except for the animal effect. These effects were estimated on the whole dataset using a model with all random effects, i.e., including the animal effect (Eq. (1)). Hence, adjustments were estimated from the full model, but phenotypes of the validation set were not adjusted for the animal effect.

Dispersion of the EBVs was evaluated by the regression coefficient of the adjusted phenotypes in the validation set on the EBVs estimated from the prediction set. A regression coefficient of one indicates the correct dispersion, while a value greater (smaller) than one indicates under (over) dispersion of the EBVs.

#### LR validation

Forward validation using the linear regression method [30] was also used to compare the prediction accuracy and dispersion of the different trait definitions. The dataset including all lines was divided into two parts; a training set, containing animals born between 2021 May and 2022 April and a validation set, containing animals born between 2022 May and 2022 October. Breeding values of the validation animals were estimated both with the complete dataset (${\mathbf{E}\mathbf{B}\mathbf{V}}_{\mathbf{c}}$) and with the partial dataset, i.e., without the records of the validation animals (${\mathbf{E}\mathbf{B}\mathbf{V}}_{\mathbf{p}}$). To compare the different trait definitions with regard to variance component and breeding value estimation, the following three metrics were computed for the validation animal: prediction accuracy, bias, and dispersion following the methods described in [30] Eqs. (4) to (6). 

4 $${\text{accuracy}} = { }\sqrt {\frac{{{\text{cov}}\left( {{\mathbf{EBV}}_{{\mathbf{p}}} ,{ }{\mathbf{EBV}}_{{\mathbf{c}}} } \right)}}{{\left( {1 - {\overline{\text{F}}}} \right)\widehat{{{\upsigma }_{{\text{a}}}^{2} }}}}}$$

5 $${\text{bias}} = { }\overline{{{\text{EBV}}_{{\text{p}}} }} - \overline{{{\text{EBV}}_{{\text{c}}} }}$$

6 $${\text{dispersion}}\;{\text{factor}} = { }\frac{{{\text{cov}}\left( {{\mathbf{EBV}}_{{\mathbf{p}}} , {\mathbf{EBV}}_{{\mathbf{c}}} } \right)}}{{{\text{var}}\left( {{\mathbf{EBV}}_{{\mathbf{p}}} } \right)}}$$

where $\widehat{{\upsigma }_{\text{a}}^{2}}$ is the estimate of the additive genetic variance based on the univariate models and $\overline{\text{F} }$ is the average inbreeding coefficient of the validation animals, calculated as the mean of the diagonal elements of the pedigree relationship matrix minus one. In addition to the joint analysis across all lines mentioned here, we also estimated the metrics from Eqs. (4) to (6) for the four lines with the most data separately.

### Selection intensity

As the traits in our study are either binary or have a limited number of levels, we expected some deviations of the EBVs from normality. Such deviations from normality can lead to realized selection intensities (i.e., the deviation of the mean of the standardized EBVs from the population mean) that deviate from the those calculated based on selected proportions as tabulated, e.g. in [31]. To illustrate this issue, imagine damage as a binary quantitative trait with a prevalence of p = 0.1. Suppose we select against this trait simply based on an individual’s own phenotype. Then we cannot differentiate among the top 90% of the individuals, i.e., the animals that show no damage. Hence, all selected proportions smaller than 0.9 will result in the same selection differential as all the top 90% of the animals have the same phenotype. Thus, the selection intensity will be much lower for this binary trait than for a trait with a more continuously distributed phenotype. While the EBVs for such traits will be less discrete than the phenotypes, the discrete nature of phenotypes can carry over to the EBVs to some degree, and the consequences for selection intensity may differ between damage trait definitions. To take this issue into account, we derived the realized selection intensities when selecting the top 5% based on the EBV for each trait by dividing the resulting selection differential, computed as the difference in average EBV of the top 5% from that of all selection candidates, by the standard deviation of the EBV. For this purpose, we used the EBV from the univariate models in Eq. (1) adjusted for the herd-year-season of scoring in an additional linear model to adjust for genetic trend. We derived the realized selection intensity by combining all lines in a joint analysis and also for the four lines with the most data available separately.

### Response to selection

To compare trait definitions in terms of response to selection, accounting for differences in both accuracy and selection intensity, we calculated the expected direct and correlated responses to selection per generation for each trait definition (Table 2). We predicted genetic gains in genetic standard deviation units to facilitate comparison between traits. To calculate the genetic gain, we used the realized selection intensities for 5% selected, as derived previously, estimates of the additive genetic standard deviations from the univariate models, and the accuracies from the LR cross-validation. The accuracies from the LR validation method were used as they represent forward cross-validation which is relevant in breeding programs. Responses to selection were derived following:

$${\text{R}}_{{{\text{direct}}}} = {\text{i}}_{{{\text{direct}}}} {\text{*r}}_{{{\text{direct}}}}$$

$${\text{R}}_{{{\text{correlated}}}} = {\text{R}}_{{{\text{direct}}}} {\text{*r}}_{{\text{g}}}$$

where ${\text{R}}_{\text{direct}}$ is the direct response, i.e. the response in the trait that is being selected for, ${\text{R}}_{\text{correlated}}$ is the indirect response, i.e. the response in the correlated traits, ${\text{i}}_{\text{direct}}$ is the selection intensity for the selected trait, ${\text{r}}_{\text{direct}}$ is the accuracy of the selected trait, and ${\text{r}}_{\text{g}}$ is the estimate of the genetic correlation between the traits from the across-line models. Note that genetic standard deviations were left out of the formulas because we show the results in genetic standard deviation units for comparability. In addition to the joint analysis, we also calculated the response to selection in the four lines with the most data separately.

## Results

In the following sections, we will first present the estimates of the genetic parameters for the seven trait definitions, both from the within and across-line analyses. Then, we will use model derived and cross-validation accuracy, dispersion, selection intensity, and predicted response to selection to compare the trait definitions.

### Genetic parameters

#### Across line analysis

Table 3 shows the estimates of heritabilities and genetic and phenotypic correlations for the seven trait definitions. The heritability estimates ranged from 0.036 to 0.061, with the lowest estimate for TD01 and the highest for SD06. Heritability estimates were larger for the original 0–3 scale and for the summed traits than for the binary traits, but these differences were not statistically significant. The phenotypic variances for the traits were estimated by summing the estimates for all the variance components, including herd-year-season of scoring. Other studies might choose to include herd-year-season effects as fixed and therefore exclude this variance from the phenotypic variance. To aid comparison with future studies, heritability estimates where the herd-year-season of scoring is left out of the phenotypic variance can be found in Additional file 1 Table S1. Estimates of the genetic correlation between ear damage (ED01 and ED03) and tail damage (TD01 and TD03) ranged from 0.41 and 0.45, i.e. these two traits are moderately correlated. Estimates of the genetic correlation between ear damage and the summed traits (AD01, NBP, and SD06) were high (> 0.83). The estimates of the genetic correlation between tail damage and the summed traits were slightly lower (> 0.73). The estimate of the genetic correlation between the binary and the original scale traits was 0.97 for ear damage and 0.85 for tail damage. The estimates of the genetic correlation between the summed traits were greater than 0.95. Estimates of phenotypic correlations were lower than those of genetic correlations, with one exception (TD03-TD01).

**Table 3:** Estimates of genetic parameters and their standard errors for all the trait definitions from the across-line analyses

	ED03	ED01	TD03	TD01	AD01	NBP	SD06
**ED03**	0.050 (0.007)	0.971 (0.010)	0.444 (0.099)	0.409 (0.103)	0.835 (0.040)	0.846 (0.036)	0.893 (0.026)
**ED01**	0.909 (0.001)	0.044 (0.007)	0.448 (0.103)	0.418 (0.106)	0.847 (0.036)	0.876 (0.030)	0.880 (0.032)
**TD03**	0.088 (0.007)	0.089 (0.007)	0.046 (0.008)	0.849 (0.033)	0.808 (0.050)	0.779 (0.052)	0.800 (0.043)
**TD01**	0.083 (0.008)	0.093 (0.008)	0.878 (0.009)	0.036 (0.006)	0.824 (0.043)	0.804 (0.044)	0.730 (0.056)
**AD01**	0.685 (0.004)	0.755 (0.004)	0.578 (0.005)	0.675 (0.004)	0.048 (0.007)	0.994 (0.002)	0.960 (0.014)
**NBP**	0.698 (0.004)	0.769 (0.003)	0.617 (0.004)	0.708 (0.004)	0.976 (0.003)	0.051 (0.007)	0.952 (0.013)
**SD06**	0.798 (0.003)	0.731 (0.003)	0.670 (0.004)	0.581 (0.006)	0.856 (0.003)	0.889 (0.003)	0.061 (0.008)

Diagonals are the heritabilities, above the diagonals are the genetic correlations, and below the diagonals are the phenotypic correlations. Trait definitions are as in Table 2. Note that the values for the two bivariate models (AD01-NBP and TD01–TD03) were estimated while fixing the correlation for litter effects at 0.99 to help convergence

#### Within line analysis

Table 4 shows estimates of heritabilities based on the within-line analyses. The estimates ranged from 0.00 to 0.10. Traits and lines with higher incidences tended to have higher heritability estimates (compare Table 4 and Fig. 2). The magnitude of the within-line heritability estimates were in line with those of of the across-line analyses but the standard errors of the estimates were larger for the within-line analyses. The estimates for multilevel traits were generally lower or similar to the corresponding estimates for the binary traits.

**Table 4:** Estimates of heritabilities and their standard errors based on the within-line analysis

	Dam line 1	Dam line 2	Dam line 3	Sire line 1	Sire line 2	Sire line 3
ED03	0.071 (0.020)	0.050 (0.043)	0.024 (0.008)	0.014 (0.024)	0.010 (0.006)	–
ED01	0.077 (0.021)	0.050 (0.043)	0.024 (0.008)	0.014 (0.024)	0.018 (0.008)	–
TD03	0.044 (0.014)	0.023 (0.035)	0.010 (0.006)	0.010 (0.031)	0.030 (0.009)	0.096 (0.057)
TD01	0.045 (0.015)	0.028 (0.034)	0.015 (0.007)	0.001 (0.023)	0.039 (0.009)	0.051 (0.046)
AD01	0.083 (0.021)	0.063 (0.043)	0.015 (0.007)	0.009 (0.024)	0.043 (0.011)	0.040 (0.046)
NBP	0.095 (0.023)	0.080 (0.044)	0.025 (0.009)	0.009 (0.024)	0.033 (0.009)	0.027 (0.044)
SD06	0.086 (0.022)	0.070 (0.044)	0.026 (0.009)	0.005 (0.023)	0.020 (0.007)	0.099 (0.058)

Trait definitions are as in Table 2. Note that in sire line 3 the prevalence of ear damage was very low (5/1663), therefore no genetic parameters were estimated for the ear damage traits

The results of within-line bivariate analyses of the four largest lines are in Additional file 1 Tables S2 to S5. Some of these analyses showed convergence problems, as indicated in the corresponding tables, and, therefore, some components were constrained. Estimates of the genetic correlation between ear damage and tail damage traits ranged from 0.29 to 0.67 for the lines for which estimates were obtained (dam line 1, dam line 3, and sire line 2). Whether ear or tail damage was more correlated with the summed damage traits differed between lines.

### Effect of sex

A significant interaction between line and sex was found both for ear and tail damage. To study this effect, we fitted the model (Eq. (1)) separately for each line. For tail damage, only sire line 3 showed a significant sex effect (p < 0.001), with gilts having less tail damage than boars, with an estimated effect of − 0.181 on the 0–3 scale (TD03) and − 0.087 on a binary scale (TD01). For ear damage, only dam line 3 showed a significant sex effect (p < 0.001), with gilts having more ear damage than boars, with an estimated effect of 0.214 on the 0–3 scale (ED03) and 0.076 on a binary scale (ED01). Based on these results, we conclude that the effect of sex on ear and tail damage is line dependent.

### Effect of age at scoring

The linear effect of age at scoring was significant for all traits, except for ED03, for which it was nearly significant (p = 0.051). Table 5 shows the estimates of the age effect for each trait definition, with the corresponding standard errors. We also fitted a quadratic effect of age at scoring on the damage traits. However, because the differences between the quadratic and linear models were minimal for the range of scoring ages in our dataset and the estimates of genetic parameters did not change, we kept the model with a linear effect.

**Table 5:** Effect of age at scoring on the different trait definitions

	ED03	ED01	TD03	TD01	AD01	NBP	SD06
Effect (s.e.)	0.00107 (0.0005)	0.00076 (0.00023)	0.00126 (0.00041)	0.00068 (0.00021)	0.00113 (0.00029)	0.00139 (0.00033)	0.00224 (0.00067)

Trait definitions are as in Table 2. The effects in the table refer to the estimated increase in the damage score per increase in age at scoring in days. The results shown here are based on combining all lines in one analysis

### Validation

#### Model derived accuracy

**Fig. 3:**
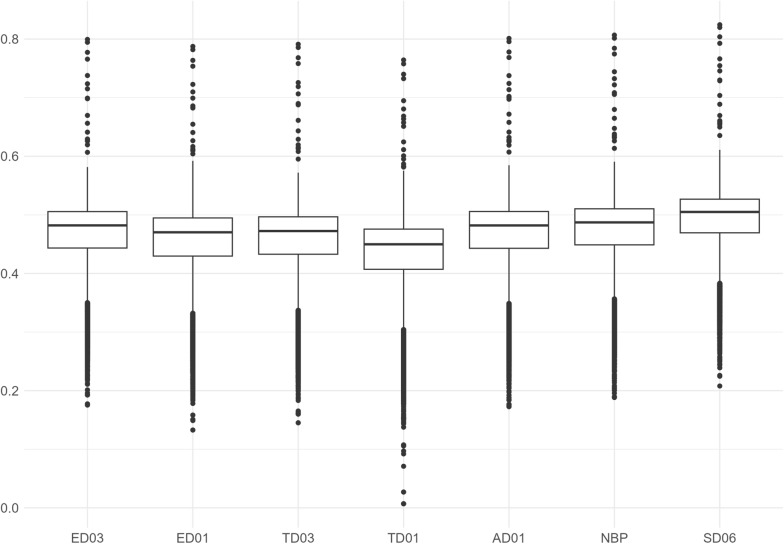
Boxplots of model derived accuracies by trait. Trait definitions are as in Table 2. Values that are 1.5 times the inter quartile range lower (higher) than the first (third) quartile are shown as outliers. The results shown here are based on combining all lines in one analysis

The mean accuracies of EBVs based on the reported standard errors from the univariate models ranged from 0.430 to 0.488. Model derived accuracies were lowest for TD01 and highest for SD06 (see boxplots in Fig. 3).

#### Five-fold cross-validation

Table 6 shows the mean accuracies and dispersion estimated based on the five-fold cross-validation with random group assignment. The mean accuracy ranged from 0.409 to 0.572 and the mean dispersion ranged from 0.974 to 1.038. ED03 had the highest accuracy, followed by SD06. The tail damage traits (TD03 and TD01) had the lowest accuracy. The mean accuracies were lowest for the binary definitions. The mean dispersion was close to one for all traits, so the EBVs were not under- or overdispersed. The results of cross-validation scenarios based on grouping full-sibs or half-sibs into the same prediction and validation groups are in Additional file 1, Tables S6-7. The mean accuracy ranged from 0.357 to 0.539 for the full-sib grouping and from 0.142 to 0.371 for the half sib grouping. Most of the traits showed an overdispersion of EBVs with the half-sib grouping.

**Table 6:** Mean accuracies and dispersions and their standard errors based on 100 replicates of five-fold cross-validation

	Accuracy	Dispersion
ED03	0.572	1.038
ED01	0.502	1.008
TD03	0.415	0.975
TD01	0.409	0.974
AD01	0.469	0.988
NBP	0.484	0.993
SD06	0.541	1.016

Trait definitions are as in Table 2. All standard errors were smaller than 0.01. The results shown here are based on combining all lines in one analysis

#### LR validation

In the forward validation, the bias was negligible and ranged from − 0.042 to 0.024 genetic standard deviation units, the accuracy ranged from 0.273 to 0.357, and the dispersion ranged from 0.907 to 1.178 (Table 7). SD06 had the highest accuracy and TD01 the lowest. For all trait definitions, the accuracies were lower for the binary trait definitions than their multilevel equivalents. Estimates for bias, accuracy, and dispersion for the lines with more data are in Additional file 1 Tables S8–11.

**Table 7:** Results from forward validation

	Bias	Accuracy	Dispersion
ED03	− 0.042	0.350	1.178
ED01	− 0.040	0.318	1.112
TD03	0.024	0.281	0.985
TD01	0.019	0.273	0.907
AD01	− 0.025	0.305	0.949
NBP	− 0.016	0.317	0.975
SD06	− 0.021	0.357	1.071

Trait definitions are as in Table 2. Bias is expressed in genetic standard deviation units. The results shown here are based on combining all lines in one analysis

### Selection intensity

Table 8 shows the realized selection intensities for a lower-tail selected proportion of 5% based on combining all the lines. For 5% selected, the selection intensity based on the standard normal distribution is 2.063. For three traits (ED03, ED01, and SD06) the realized selection intensity was greater than the tabulated value. This is due to a positive excess kurtosis of the distribution of the EBVs of these traits. For the other traits, the realized selection intensities were lower than the tabulated values. Table 8 also shows that binary traits had lower selection intensities than their more continuous counterparts. Realized selection intensities for within-line analyses for the lines with more data available are in Additional file 1 Table S12. Note that direct comparison of selection intensities for the binary and multilevel traits is not always straightforward, as the multilevel trait might lead to a distribution with a longer upper tail, which leads to fewer animals in the lower tail of the distribution. This effect was most visible for the tail damage traits in sire line 3, which had better differentiation of estimated breeding values on the right side of the distribution for TD03. However, since the desired direction of selection is towards less damage, the selection intensity was higher for the binary trait TD01 in this line.

**Table 8:** Realized selection intensities based on the actual distribution of EBVs for a lower-tail selected proportion of 5%

Trait	ED03	ED01	TD03	TD01	AD01	NBP	SD06
Intensity	2.237	2.071	1.904	1.788	2.036	1.963	2.137

Trait definitions are as in Table 2. The corresponding selection intensity from the standard normal distribution is 2.063. The results shown here are based on combining all lines in one analysis

### Response to selection

Table 9 shows the predicted direct and correlated responses to selection, expressed in genetic standard deviation units. To calculate these, we used the following estimates from the across-line analyses: realized selection intensities from Table 8, accuracies from the forward validation of Table 7, and the estimated genetic correlations from Table 3. For the ear damage traits, selection on ED03 showed the highest response. For all other traits, selection on SD06 showed the highest response. This shows that the differences in accuracy and selection intensity, in combination with high genetic correlations, favours indirect selection for most of the studied traits. Estimates of responses to selection in the four lines with the most data are in Additional file 1 Tables S13 to 16.

**Table 9:** Predicted direct and correlated response to selection

	Selection trait							
		ED03	ED01	TD03	TD01	AD01	NBP	SD06
**Response trait**	**ED03**	**0.783**	0.639	0.237	0.199	0.519	0.527	0.681
	**ED01**	**0.760**	0.658	0.240	0.204	0.526	0.545	0.671
	**TD03**	0.347	0.295	0.535	0.414	0.502	0.485	**0.610**
	**TD01**	0.320	0.275	0.454	0.488	0.512	0.501	**0.557**
	**AD01**	0.654	0.558	0.432	0.402	0.621	0.618	**0.732**
	**NBP**	0.663	0.577	0.417	0.393	0.617	0.622	**0.726**
	**SD06**	0.699	0.579	0.428	0.356	0.596	0.592	**0.763**

Selection was based on the traits mentioned in the columns. The rows show the response in all the traits. For example 0.760 is the correlated response in ED01 when selecting on ED03. The diagonals are direct responses, and the off diagonals are correlated responses. Trait definitions are as in Table 2.The highest value per row is bold. The results shown here are based on combining all lines in one analysis

## Discussion

### Heritability

In this study, we have shown that ear and tail damage traits are lowly heritable, with heritability estimates ranging from 0.04 to 0.06 (Table 3), when all lines were analysed jointly, and from 0.00 to 0.10 for the within-line analyses (Table 4). These estimates are similar to those reported for binary tail damage in the Tai Zumu line (a Meishan-Large White composite line) [23, h^2^ = 0.06], for binary tail damage in purebred German Landrace and German Landrace x Pietrain crosses from different models [32, h^2^ = 0.01–0.46], and based on medication reports for tail biting injuries in a Large White population [24, h^2^ = 0.09]. Our heritability estimates are much smaller than estimates of ear (h^2^ = 0.46) and tail (h^2^ = 0.16) damage reported by Gorssen et al. [25], possibly due to the difference in the setup of the two studies. In the study of Gorssen et al. [25], individuals were evaluated multiple times for ear and tail damage throughout the finishing phase and the pens were composed of full- or half-sibs. In such a setup, the heritability estimate also captures part of the social genetic variance [33, 34] and, as a result, their heritability estimates are expected to be between the direct heritability (h^2^) and the total heritable variance as a proportion of the phenotypic variance (T^2^) [35].

### Genetic correlations

The estimates of genetic correlations between tail and ear damage based on the across-line analyses ranged from 0.41 to 0.45 (Table 3), meaning that these are different traits. Although the genetic correlation estimates varied largely between lines in the within-line analyses, positive estimates were also observed in the lines with most data available (see Additional file 1 Tables S2 to S5). Gorssen et al. [25] reported a genetic correlation between ear and tail damage of a similar magnitude (0.32). However, we emphasize that the genetic difference between ear and tail damage, both scored at the receiver level, does not imply that ear and tail biting behaviours themselves are genetically different traits. In other words, the genetic propensity to perform tail vs. ear biting might still be very similar. Some studies have reported that pigs that display more tail biting also perform more ear biting [27, 36]. Goossens et al. [37] showed that more ear biting behaviour occurred on farms with pigs with shortly docked tails, indicating that the motivation to perform the behaviour could be similar for ear and tail biting. Future studies on large scale behaviour recordings could answer whether tail biting and ear biting indeed have a similar genetic basis.

### Phenotype definition

In this paper, we assume that damage is the result of harmful behaviour performed by pen mates. However, the phenotype we observe is a combination of different origins of damage, as we also included necrosis. Necrosis can occur independent of harmful behaviour (reviewed by [20, 38]). Furthermore, necrosis is hypothesised to promote biting behaviour, as the victims might find it relieving when other pigs manipulate their lesions [39]. Therefore, the cause and effect relationship between biting behaviour and damage is not fully clear and may go in both directions. In addition, ears or tails can occasionally also be damaged for other reasons.

### Effect of sex on damage

Studies comparing the occurrence of tail damage in female and intact male pigs, either in mixed-sex or single-sex groups have yielded inconsistent results [5, 40]. In this study, sex affected tail damage in one (boar) line only, with higher damage scores for boars than for gilts. This boar line contained both mixed-sex and single-sex groups and it is, therefore, difficult to disentangle who the biters were. We tested pen composition as a fixed effect, but it was not significant and left out of the final models. Conversely, in one of the dam lines, ear damage was higher for gilts than for boars. In other lines, the sex effect on ear damage was not significant.

### Comparison of trait definitions

One of the aims of this study was to compare trait definitions to help breeders decide which trait(s) to measure and obtain EBVs for. We base our recommendations on the assumption that ED03 and TD03 are the traits in the breeding goal and, therefore the traits for which genetic gain should be maximized. This was based on the assumption that the difference between the phenotypes 1 and 3 on the original scale for ear and tail damage is relevant for animal welfare and this difference between levels would be lost when using binary trait definitions.

The across-line analyses showed that multilevel traits are superior compared to their binary counterparts with regard to heritability, accuracy, and selection intensity. However, for breeders, the most important criterion is response to selection. Table 9 shows that the highest response in ED03 can be achieved by direct selection, as ED03 had the overall highest selection intensity and its accuracy and heritability estimates were very close to those of SD06. For TD03 the highest gain was achieved by selecting on the summed trait SD06. For TD03 indirect selection based on SD06 outperformed direct selection because TD03 had lower accuracy, heritability, and selection intensity than SD06 and it also had a high genetic correlation with SD06. The response in ED03 when selecting on SD06 was 87% of the response possible when selecting on the trait itself. Thus, selecting on SD06 instead of ED03 might be satisfactory for breeding companies, especially if there is interest to limit the number of traits in the genetic evaluation. In conclusion, in case only one trait is chosen for genetic evaluation, we recommend using SD06. However, selection on the summed trait SD06 does not allow breeders to weigh traits independently in an index. To optimize response in both tail and ear damage, EBVs for both traits should ideally be included in a weighted index, rather than selecting for the summed records for both traits. In addition, the weights for ear and tail damage could be different if they are only based on economic values. Tail damage is associated with carcass condemnation [8–10, 41], resulting in a loss of revenue and, therefore, has a higher economic value than ear damage. However, as the damage traits in question are welfare traits, their weights should not be based on profit only. The objective of this paper was to investigate how the summed traits behave in comparison to the individual traits, rather than giving recommendations about index weights on ear damage versus tail damage.

### Current and future phenotyping

The phenotyping protocol used in this study was on a 0 to 3 scale, measured at the end of the testing phase. This scale allows for quick phenotyping on farms. We used this scale and also created some combinations and simplifications of it to compare trait definitions. When using a linear model for phenotypes on the 0 to 3 scale, we assumed that the difference between scores 0 and 1 is the same as between 1 and 2, which may not be correct from a welfare point. Refining the scale with a more strict phenotyping protocol could make the scale more fair and linear. However, this refinement could make the phenotyping more difficult, and as a result, no longer be feasible in practice. Computer vision could provide an alternative that requires less labour. In that case, not just the victims but also the performers of harmful behaviour could be identified, and longitudinal data throughout the testing phase could be obtained. Some developments have already been made in this area. For example, an algorithm is available for tail biting detection when the victim reacts by moving away [42]. However, tail biting can result in different reactions of the victims and often victims do not visibly react at all (see ethogram in [36]). Therefore, future algorithms must be able to detect tail biting regardless of the victim’s reaction. Apart from behaviour detection, (re)identification of individual pigs, which is required for phenotyping, is challenging [43].

### Validation methods

In this study, we used two types of validation of EBV, a five-fold cross-validation and the LR forward validation. The main difference between these two methods is the way the animals are divided between the validation and the test set. In the five-fold cross-validation, all age groups can end up in the validation set, whereas in the LR method, the validation set only includes the youngest animals. As a result, animals in both sets do not have the same random pen-group or herd-year-season effects in the LR method, while this does not hold for the five-fold cross-validation. Therefore, we adjusted the validation phenotypes in the five-fold cross-validation for all random effects, except for the EBV. Results without adjustment showed strong underdispersion of EBVs (results not shown). Furthermore, we used the full genetic model to estimate the random effects we adjusted for, to minimize the confounding of especially the pen-group effect, which explained the most variance, and the genetic effect. A similar method of correcting for random effects in the validation phenotypes was used by García-Ballesteros et al. [44] in endurance horses and by Gorssen et al. [45] in pigs. This illustrates the importance of choices when adjusting the raw phenotypes for random effects in k-fold cross-validations. The comparison of the forward and the random five-fold cross-validation shows that the dispersion was close to one for both validation methods. However, the estimated accuracies were lower with the forward validation. This difference is probably due to the presence of sibs of validation individuals in the training data with random five-fold cross-validation, which is limited in the forward validation. To verify this, we ran two additional five-fold cross-validation scenarios: one where all full-sibs were in the same group (FS scenario) and one where all half-sibs (HS scenario) were in the same group (Additional file 1 Tables S6 to S7). Thus, the information for EBV in validation comes from half-sib and weaker relationships in the FS scenario and from relationships weaker than half-sib relationships in the HS scenario comes. The accuracy of the LR validation was between these two extra scenarios for all traits, except for ED03, which is reasonable as the validation and training individuals had 66 sires in common in the LR validation.

### Joint analysis of six lines

This study highlights the challenge of dealing with small datasets. Our dataset consisted of around 33 K animals from six lines, with unequal distribution of the number of animals between the lines (Table 1). We chose to combine all lines in one analysis and to correct for a line effect. Hence, we assumed that all lines had the same genetic variance. We used pedigree relationships that did not include across-line relationships. If we had used a genomic relationship matrix, then relationships between lines could have been included. However, in that case, we would have had to either assume that the SNP effects are the same for all lines or estimate the genetic correlation between the lines or between SNP effects in the different lines. Furthermore, the use of a genomic relationship may bias the heritability estimates if there is selective genotyping [46]. Therefore, we do not expect that using a genomic relationship matrix would yield more accurate variance component estimates than the ones we present here.

The current method of combining all lines assumes that the genetic parameters are the same for all lines and that there are differences only in trait means between the lines. However, lines could have different variances, as they have different selection histories. However, the within-line analyses showed only small differences in the heritability estimates between lines. Counterintuitively, in the within-line analyses, we observed lower heritability estimates for the multilevel traits compared to the binary ones, though the differences were not statistically significant. In addition, the bivariate models had convergence problems for several trait combinations, which makes drawing conclusions from the current within-line models difficult.

### Realized selection intensity

Our results show that traits differ not only in accuracy of EBV, but also in selection intensity, and that this has to be considered when choosing the optimal trait. Using traits with a few levels can result in selection intensities when selecting on EBV to differ from those expected based on the standard normal distribution. For some traits, such as ED03, the realized selection intensity was higher than expected. However, for other traits, like TD03, the realized selection intensity and thereby the potential to improve the trait was lower than expected. Realized selection intensities that are higher than expected are in our case probably an artefact of treating the lines as one population with a single genetic variance. Based on EBV derived using the within-line analyses, the realized intensities never exceeded the theoretical expectation, although the differences between trait definitions were also present within the lines. However, the differences in realized selection intensities between traits of different number of levels, i.e. binary or multilevel, were not large and not always consistent with the across-line analysis. Whether the multilevel or the binary trait has a higher selection intensity is probably dependent on the prevalence of damage. In our study, selection was towards the less differentiated side of the trait, i.e. towards no damage. In case the direction of selection is towards the more differentiated side of the scale, the comparison of selection intensities of binary and multilevel traits might give different results.

### Social genetic effects

An important aspect that this study has not taken into account is the role of social or indirect genetic effects on the evaluated traits. The damage phenotypes we measured here are the result of an interaction between pigs and are thus affected by both the tendency of the victim to be bitten and by the tendency of pen mates to perform biting. Therefore, two genetic effects that affect tail and ear damage can be distinguished, a direct genetic effect (DGE) and a social genetic effect (SGE) [47, 48]. The DGE represents the effects of the genes of the focal individual (i.e. the victim) on the damage of its own tail and ears, whereas the SGE represents the genetic effects of the pen mates on the damage phenotype of the focal individual, which reflects the damaging behaviour of the pen mates. Social genetic models are a statistical solution to evaluate the biting behaviour without having observations on the actors themselves. Past studies have used social genetic effect models to estimate genetic parameters of harmful behaviours. For example, Leite et al. [49] used a binary skin damage score, Angarita et al. [50] looked at skin lesion count 24 h post mixing, and Canario & Flatres-Grall [23] used binary tail damage. In the models used in the current study, the SGE are mostly captured by the random pen-group effect, as the pen-group effect also absorbs the social environment that the pen mates share. Thus, we do not expect that the presence of SGE will have a had large effect on the estimates for DGE. Nevertheless, we did not capture the heritable variance in tail and ear damage due to SGE in the current analysis. In a future study, we will include SGE, to estimate the total heritable variation that is available for breeding for lower tail and ear damage. The use of social genetic models will also allow us to estimate the genetic correlation between the direct and the social genetic effect (e.g., [51]), which quantifies whether selecting on the DGE also leads to a correlated response in the SGE. In case of a positive correlation, selecting against damage would also reduce the harmful behaviour. In case of a negative correlation, however, individual selection against damage using groups composed at random with respect to relatedness is expected to increase the harmful behaviour [51, 52].

## Conclusions

Tail and ear damage in pigs are heritable traits with heritability estimates ranging from 0.04 to 0.06 based on the across-line models. Tail and ear damage were estimated to have a moderate genetic correlation, ranging from 0.41 to 0.45 based on the across-line models, indicating that they are genetically distinct. The accuracy of EBVs was moderate and mostly higher for the traits on the original 0–3 scale compared to their binary versions. The dispersion error of the EBVs was minor and there was no evidence of bias with the current models. For ear damage on the original scale the highest response to selection can be expected when selecting on the trait itself, whereas for tail damage on the original scale, selection on summed damage showed the highest gain. Altogether, results from this study show that genetic improvement of the direct genetic effect of ear and tail damage is possible.

## Supplementary Information

**Additional file 1: Table S1.** Heritability without including herd-year-season of scoring. **Table S2.** Genetic parameters for dam line 1. **Table S3.** Genetic parameters for dam line 3. **Table S4.** Genetic parameters for sire line 2. **Table S5.** Genetic parameters for sire line 3. **Table S6.** Results of cross-validation with full-sib grouping. **Table S7.** Results of cross-validation with half-sib grouping. **Table S8.** LR metrics for dam line 1. **Table S9.** LR metrics for dam line 3. **Table S10.** LR metrics for sire line 2. **Table S11.** LR metrics for sire line 3. **Table S12.** Selection intensity for the four most frequent lines. **Table S13.** Response to selection in dam line 1. **Table S14.** Response to selection in dam line 3. **Table S15.** Response to selection in sire line 2. **Table S16.** Response to selection in sire line 3.

## Data Availability

The data is the property of Topigs Norsvin and cannot be shared.
