# Supplementary material for: Genetic parameters and potential of reducing tail and ear damage in pigs through breeding
Source: Genet Sel Evol. 2025 Jul 14;57:39. doi: 10.1186/s12711-025-00976-0 (PMC12257756; doi:10.1186/s12711-025-00976-0)
Supplement: Supplementary file 1 — Additional file 1: Table S1. Heritability without including herd-year-season of scoring. Table S2. Genetic parameters for dam line 1. Table S3. Genetic parameters for dam line 3. Table S4. Genetic parameters for sire line 2. Table S5. Genetic parameters for sire line 3. Table S6. Results of cross-validation with full-sib grouping. Table S7. Results of cross-validation with half-sib grouping. Table S8. LR metrics for dam line 1. Table S9. LR metrics for dam line 3. Table S10. LR metrics for sire line 2. Table S11. LR metrics for sire line 3. Table S12. Selection intensity for the four most frequent lines. Table S13. Response to selection in dam line 1. Table S14. Response to selection in dam line 3. Table S15. Response to selection in sire line 2. Table S16. Response to selection in sire line 3. [file 12711_2025_976_MOESM1_ESM.pdf]

Additional file 1: Results of within-line analyses and additional 5-fold cross-validation scenarios

Description: In this file supplementary Tables S1-S16 can be found. Table S1 contains heritabilities without including herd-year-season of scoring in the phenotypic variance both for within- and across-lines analyses. Tables S2-S5 include within line genetic parameters for the four lines with the most data. Note that for sire line 3 no estimate of ear damage is provided due to the low prevalence of ear damage in this line. Tables S6-7 include the results of FS and HS scenarios of the 5-fold cross-validation. Tables S8-S11 contain the LR validation metrics for the four largest lines. Tables S12 contains the selection intensities calculated for the four largest lines. Tables S13-S16 contain the response to selection for the four largest line calculated using the line specific genetic parameters, LR accuracies, and selection intensities. Note that sometimes due to convergence problems the models did not yield any results and therefore the tables with genetic parameters (S2-S5) contain “error”. As a result the corresponding parts in the response could also not be estimated, this is noted by “missing information” in Tables S13-S16.

Additional file 1 Table S1

Title: Heritability without including herd-year-season of scoring

**trait**	**h^2 across line**	dam line 1	dam line 2	dam line 3	sire line 1	sire line 2	sire line 3
**ED03**	0.051 (0.007)	0.073 (0.020)	0.050 (0.043)	0.024 (0.008)	0.014 (0.024)	0.010 (0.006)	-
**ED01**	0.045 (0.007)	0.079 (0.021)	0.050 (0.043)	0.024 (0.008)	0.014 (0.024)	0.018 (0.008)	-
**TD03**	0.047 (0.008)	0.044 (0.015)	0.024 (0.037)	0.010 (0.006)	0.010 (0.031)	0.030 (0.009)	0.096 (0.057)
**TD01**	0.037 (0.007)	0.045 (0.015)	0.029 (0.035)	0.015 (0.007)	0.001 (0.023)	0.039 (0.010)	0.054 (0.05)
**AD01**	0.049 (0.007)	0.085 (0.021)	0.066 (0.044)	0.015 (0.007)	0.009 (0.024)	0.044 (0.011)	0.042 (0.048)
**NBP**	0.052 (0.007)	0.097 (0.023)	0.082 (0.046)	0.025 (0.009)	0.009 (0.024)	0.034 (0.009)	0.029 (0.046)
**SD06**	0.063 (0.008)	0.089 (0.022)	0.074 (0.046)	0.027 (0.009)	0.005 (0.023)	0.020 (0.007)	0.100 (0.058)

Heritabilities without including herd-year-season of scoring in the phenotypic variance both for within- and across-lines analyses. Trait definitions are as in Table 2.

Additional file 1 Table S2

Title: Genetic parameters for dam line 1

Dam line 1	**ED03**	**ED01**	**TD03**	**TD01**	**AD01**	**NBP**	**SD06**
**ED03**	0.071 (0.020)	0.935 (0.028)(c.p.)	0.530 (0.191)(c.p.)	0.543 (0.182)	0.957 (0.029)(c.p.)	0.958 (0.028)(c.p.)	0.954 (0.024)
**ED01**	0.967 (0.001)(c.p.)	0.077 (0.021)	0.485 (0.190)(c.p.)	0.462 (0.189)(c.p.)	0.938 (0.030)(c.p.)	0.952 (0.024)	0.938 (0.029)(c.p.)
**TD03**	0.053 (0.016)(c.p.)	0.054 (0.016)(c.p.)	0.044 (0.014)	0.874 (0.088)(c.p.)	0.731 (0.12)(c.p.)	0.754 (0.105)(c.p.)	0.762 (0.109)(c.p.)
**TD01**	0.048 (0.015)	0.054 (0.015)(c.p.)	0.930 (0.002)(c.p.)	0.045 (0.015)	0.762 (0.109)	0.802 (0.097)(c.p.)	0.778 (0.109)
**AD01**	0.810 (0.006)(c.p.)	0.836 (0.005)(c.p.)	0.497 (0.012)(c.p.)	0.540 (0.011)	0.083 (0.021)	0.912 (0.031)(c.p.)	0.999 (0.006)(c.p.)
**NBP**	0.806 (0.005)(c.p.)	0.829 (0.005)	0.561 (0.011)(c.p.)	0.548 (0.012)(c.p.)	0.967 (0.001)(c.p.)	0.095 (0.023)	0.946 (0.034)(c.p.)
**SD06**	0.865 (0.004)	0.839 (0.005)(c.p.)	0.547 (0.011)(c.p.)	0.510 (0.011)	0.930 (0.002)(c.p.)	0.955 (0.001)(c.p.)	0.086 (0.022)

Trait definitions are as in Table 2. Note that models where the likelihood did not converge or parameters were restrained are marked with "(c. p.)", which stands for convergence problem.

Additional file 1 Table S3

Title: Genetic parameters for dam line 3

**Dam line 3**	**ED03**	**ED01**	**TD03**	**TD01**	**AD01**	**NBP**	**SD06**
**ED03**	0.024 (0.008)	0.997 (0.017)	0.665 (0.24)	0.421 (0.247)	0.94 (0.079)	0.913 (0.063)	0.963 (0.034)
**ED01**	0.901 (0.002)	0.024 (0.008)	0.672 (0.239)	0.346 (0.256)	0.907 (0.074)	0.902 (0.056)	0.943 (0.051)
**TD03**	0.170 (0.011)	0.140 (0.011)	0.010 (0.006)	0.728 (0.170)(c.p.)	0.913 (0.126)	0.945 (0.095)	0.843 (0.119)
**TD01**	0.139 (0.011)	0.124 (0.011)	0.886 (0.002)(c.p.)	0.015 (0.007)	0.711 (0.164)(c.p.)	0.717 (0.14)	0.648 (0.167)
**AD01**	0.732 (0.005)	0.813 (0.004)	0.547 (0.008)	0.617 (0.007)(c.p.)	0.015 (0.007)	0.992 (0.010)(c.p.)	0.978 (0.155)(c.p.)
**NBP**	0.752 (0.005)	0.817 (0.004)	0.62 (0.007)	0.674 (0.006)	0.964 (0.001)(c.p.)	0.025 (0.009)	0.95 (0.144)(c.p.)
**SD06**	0.832 (0.003)	0.743 (0.005)	0.687 (0.006)	0.601 (0.007)	0.848 (0.003)(c.p.)	0.903 (0.002)(c.p.)	0.026 (0.009)

Trait definitions are as in Table 2. Note that models where the likelihood did not converge or parameters were restrained are marked with "(c. p.)", which stands for convergence problem.

Additional file 1 Table S4

Title: Genetic parameters for sire line 2

Sire line 2	**ED03**	**ED01**	**TD03**	**TD01**	**AD01**	**NBP**	**SD06**
**ED03**	0.010 (0.006)	error	0.289 (0.332)(c.p.)	0.293 (0.304)(c.p.)	0.792 (0.165)(c.p.)	0.642 (0.196)(c.p.)	0.709 (0.185)(c.p.)
**ED01**	error	0.018 (0.008)	0.636 (0.261)(c.p.)	0.505 (0.245)(c.p.)	0.896 (0.102)	0.782 (0.119)(c.p.)	0.937 (0.094)
**TD03**	0.167 (0.010)(c.p.)	0.191 (0.01)(c.p.)	0.030 (0.009)	0.999 (0.015)(c.p.)	0.955 (0.050)(c.p.)	0.969 (0.052)(c.p.)	0.917 (0.071)(c.p.)
**TD01**	0.146 (0.010)(c.p.)	0.181 (0.010)(c.p.)	0.900 (0.002)(c.p.)	0.039 (0.009)	0.916 (0.044)(c.p.)	0.938 (0.041)(c.p.)	0.928 (0.078)(c.p.)
**AD01**	0.619 (0.006)(c.p.)	0.675 (0.005)	0.716 (0.005)(c.p.)	0.793 (0.004)(c.p.)	0.043 (0.011)	0.988 (0.008)(c.p.)	0.998 (0.032)(c.p.)
**NBP**	0.653 (0.006)(c.p.)	0.727 (0.005)(c.p.)	0.743 (0.005)(c.p.)	0.807 (0.004)(c.p.)	0.959 (0.001)(c.p.)	0.033 (0.009)	0.999 (0.018)
**SD06**	0.788 (0.004)(c.p.)	0.746 (0.004)	0.739 (0.005)(c.p.)	0.663 (0.006)(c.p.)	0.870 (0.002)(c.p.)	0.910 (0.002)	0.020 (0.007)

Trait definitions are as in Table 2. Note that models where the likelihood did not converge or parameters were restrained are marked with “(c. p.)”, which stands for convergence problem.

Additional file 1 Table S5

Title: Genetic parameters for sire line 3

Sire line 3	**TD03**	**TD01**	**AD01**	**NBP**	**SD06**
**TD03**	0.096 (0.057)	0.891 (0.152)	0.991 (0.175)	0.388 (2.472)(c.p.)	0.296 (2.639)(c.p.)
**TD01**	0.789 (0.020)	0.051 (0.046)	0.341 (0.842)(c.p.)	0.170 (1.071)(c.p.)	0.854 (0.169)(c.p.)
**AD01**	0.787 (0.019)	0.839 (0.249)(c.p.)	0.040 (0.046)	0.331 (1.075)(c.p.)	0.905 (0.188)(c.p.)
**NBP**	0.775 (0.020)(c.p.)	0.837 (0.170)(c.p.)	0.983 (0.003)(c.p.)	0.027 (0.044)	0.904 (2.723)(c.p.)
**SD06**	0.996 (0.001)(c.p.)	0.787 (0.021)(c.p.)	0.789 (0.020)(c.p.)	0.784 (0.020)(c.p.)	0.099 (0.058)

Trait definitions are as in Table 2. Note that models where the likelihood did not converge or parameters were restrained are marked with “(c. p.)”, which stands for convergence problem.

Additional file 1 Table S6

Title: Results of 5-fold cross-validation with full-sib grouping

	**accuracy**	**dispersion**
**ED03**	0.539	1.027
**ED01**	0.478	1.004
**TD03**	0.357	0.903
**TD01**	0.372	0.941
**AD01**	0.435	0.969
**NBP**	0.455	0.983
**SD06**	0.501	0.995

Trait definitions are as in Table 2. All standard errors were smaller than 0.01.

Additional file 1 Table S7

Title: Results of 5-fold cross-validation with half-sib grouping

	**accuracy**	**dispersion**
**ED03**	0.371	1.015
**ED01**	0.307	0.941
**TD03**	0.169	0.646
**TD01**	0.142	0.557
**AD01**	0.222	0.725
**NBP**	0.250	0.788
**SD06**	0.329	0.931

Trait definitions are as in Table 2. All standard errors were smaller than 0.01. Note that all results in this manuscript were obtained by only having pedigree relationships, so the relationships used to predict EBVs in the current set up are weaker than half-sib relationships.

Additional file 1 Table S8

Title: LR metrics for dam line 1

	bias	accuracy	dispersion
ED03	0.038	0.521	1.086
ED01	0.064	0.512	1.092
TD03	-0.160	0.316	1.050
TD01	-0.088	0.326	0.978
AD01	0.014	0.476	1.023
NBP	0.010	0.500	1.077
SD06	-0.031	0.521	1.106

Trait definitions are as in Table 2. Bias is expressed in genetic standard deviation units.

Additional file 1 Table S9

Title: LR metrics for dam line 3

	bias	accuracy	dispersion
ED03	-0.097	0.224	1.043
ED01	-0.077	0.259	1.009
TD03	0.039	0.173	1.055
TD01	0.037	0.186	1.007
AD01	-0.039	0.204	0.89
NBP	-0.034	0.21	0.892
SD06	-0.039	0.207	0.954

Trait definitions are as in Table 2. Bias is expressed in genetic standard deviation units.

Additional file 1 Table S10

Title: LR metrics for sire line 2

	bias	accuracy	dispersion
ED03	-0.021	0.152	0.882
ED01	-0.002	0.173	0.903
TD03	0.020	0.262	0.938
TD01	0.020	0.255	0.802
AD01	0.006	0.259	0.872
NBP	0.004	0.240	0.875
SD06	0	0.218	0.961

Trait definitions are as in Table 2. Bias is expressed in genetic standard deviation units.

Additional file 1 Table S11

Title: LR metrics for sire line 3

	bias	accuracy	dispersion
TD03	0.007	0.135	1.096
TD01	0.079	0.138	1.568
AD01	0.071	0.124	1.523
NBP	0.058	0.107	1.547
SD06	0.007	0.134	1.085

Trait definitions are as in Table 2. Bias is expressed in genetic standard deviation units.

Additional file 1 Table S12

Title: Selection intensity for the four most frequent lines

trait	Dam line 1	Dam line 3	Sire line 2	Sire line 3
ED03	1.959	1.793	1.609	Low prevalence
ED01	1.933	1.786	1.792	Low prevalence
TD03	1.640	1.736	1.563	1.517
TD01	1.647	1.876	1.548	1.950
AD01	1.893	1.826	1.762	1.917
NBP	1.853	1.748	1.890	1.880
SD06	1.863	1.657	1.845	1.507

Trait definitions are as in Table 2.

Additional file 1 Table S13

Title: Response to selection in dam line 1

	**Selection Trait**							
**Response trait**		ED03	ED01	TD03	TD01	AD01	NBP	SD06
	ED03	**1.020**	0.925	0.274	0.291	0.863	0.889	0.925
	ED01	0.954	**0.989**	0.251	0.248	0.846	0.883	0.909
	TD03	0.540	0.480	0.518	0.469	0.659	0.699	**0.739**
	TD01	0.554	0.457	0.453	0.536	0.687	0.744	**0.755**
	AD01	**0.977**	0.928	0.379	0.409	0.901	0.846	0.969
	NBP	**0.978**	0.942	0.391	0.430	0.822	0.927	0.917
	SD06	**0.973**	0.927	0.395	0.417	0.900	0.877	0.970

Trait definitions are as in Table 2. Selection was based on the traits mentioned in the columns. The rows show the response in all the traits. The diagonals are direct responses, and the off diagonals are correlated responses. Note that the models for the within-line bivariate models had several convergence problems.

Additional file 1 Table S14

Title: Response to selection in dam line 3

	**Selection Trait**							
**Response trait**		ED03	ED01	TD03	TD01	AD01	NBP	SD06
	ED03	0.401	**0.461**	0.199	0.147	0.350	0.334	0.331
	ED01	0.400	**0.462**	0.201	0.121	0.338	0.330	0.324
	TD03	0.267	0.311	0.300	0.254	0.340	**0.346**	0.290
	TD01	0.169	0.160	0.218	**0.348**	0.265	0.263	0.223
	AD01	0.377	**0.420**	0.274	0.248	0.372	0.363	0.336
	NBP	0.366	**0.417**	0.283	0.250	0.369	0.366	0.327
	SD06	0.386	**0.436**	0.253	0.226	0.364	0.348	0.344

Trait definitions are as in Table 2. Selection was based on the traits mentioned in the columns. The rows show the response in all the traits. The diagonals are direct responses, and the off diagonals are correlated responses. Note that the models for the within-line bivariate models had several convergence problems.

Additional file 1 Table S15

Title: Response to selection in sire line 2

	**Selection Trait**							
**Response trait**		ED03	ED01	TD03	TD01	AD01	NBP	SD06
	ED03	0.245	missing information	0.118	0.116	**0.361**	0.291	0.285
	ED01	missing information	0.310	0.261	0.199	**0.408**	0.354	0.377
	TD03	0.071	0.197	0.410	0.394	0.435	**0.439**	0.369
	TD01	0.072	0.156	0.409	0.395	0.417	**0.425**	0.373
	AD01	0.194	0.278	0.391	0.361	**0.456**	0.447	0.402
	NBP	0.157	0.242	0.397	0.370	0.450	**0.453**	0.402
	SD06	0.174	0.290	0.376	0.366	**0.455**	0.452	0.402

Trait definitions are as in Table 2. Selection was based on the traits mentioned in the columns. The rows show the response in all the traits. The diagonals are direct responses, and the off diagonals are correlated responses. Note that the models for the within-line bivariate models had several convergence problems.

Additional file 1 Table S16

Title: Response to selection in sire line 3

	Selection Trait							
**Response trait**	Sire line 3	ED03	ED01	TD03	TD01	AD01	NBP	SD06
	ED03	missing information	missing information	missing information	missing information	missing information	missing information	missing information
	ED01	missing information	missing information	missing information	missing information	missing information	missing information	missing information
	TD03	missing information	missing information	0.204	**0.240**	0.236	0.078	0.06
	TD01	missing information	missing information	0.182	**0.269**	0.081	0.034	0.172
	AD01	missing information	missing information	0.202	0.092	**0.239**	0.067	0.182
	NBP	missing information	missing information	0.079	0.046	0.079	**0.202**	0.182
	SD06	missing information	missing information	0.061	**0.230**	0.216	0.183	0.202

Trait definitions are as in Table 2. Selection was based on the traits mentioned in the columns. The rows show the response in all the traits. The diagonals are direct responses, and the off diagonals are correlated responses. Note that the models for the within-line bivariate models had several convergence problems.
